# New insights into the interplay between MALAT1 and miRNA-155 to unravel potential diagnostic and prognostic biomarkers of Behçet’s disease

**DOI:** 10.1007/s10067-024-07291-x

**Published:** 2025-01-11

**Authors:** Noha H. Sayed, Olfat G. Shaker, Mai A. Abd‑Elmawla, Ahmed Gamal, Nevine Fathy

**Affiliations:** 1 Biochemistry Department, Faculty of Pharmacy, Cairo University, Cairo, 11562 Egypt; 2 Medical Biochemistry and Molecular Biology Department, Faculty of Medicine, Cairo University, Cairo, 11562 Egypt; 3 Andrology, Sexology and STI’s Department, Faculty of Medicine, Cairo University, Cairo, 11562 Egypt

**Keywords:** Behçet’s disease, CD106, Disease activity, IL-6, Non-coding RNA, TNFα

## Abstract

The current study was deployed to evaluate the role of metastasis-associated lung adenocarcinoma transcript 1 (MALAT1) and miR-155, along with the inflammatory markers, TNFα and IL-6, and the adhesion molecule, cluster of differentiation 106 (CD106), in Behçet’s disease (BD) pathogenesis. The study also assessed MALAT1/miR-155 as promising diagnostic and prognostic biomarkers for BD. The current retrospective case–control study included 74 Egyptian BD patients and 50 age and sex-matched controls. Blood samples were collected, and then, serum samples were separated for further biochemical and molecular investigations. The gene expression of MALAT1 and miR-155 was measured using qRT-PCR, whereas the levels of TNFα, IL-6, and CD106 were estimated using ELISA technique. MALAT1 was significantly downregulated, whereas miR-155 was upregulated among BD patients, compared with control subjects. Levels of TNFα, IL-6, and CD106 were elevated in BD patients. Further downregulation in MALAT1 together with upregulation of miR-155 was observed in active BD patients, relative to the inactive group. Receiver-operating-characteristic analysis revealed that MALAT1 and miR-155 could discriminate BD patients from controls, on the one hand, and active from inactive BD patients, on the other hand. MALAT1 was negatively correlated with TNFα, IL-6, and CD106, while miR-155 was positively correlated with them. Logistic regression analyses demonstrated miR-155 as a significant independent predictor of BD susceptibility, and MALAT1 as an independent negative predictor of BD activity. For the first time, the current research enlightens the role of MALAT1 and miR-155 in BD pathogenesis via impacting IL-6/TNF-α/CD-106 signaling. As well, MALAT1 and miR-155 could be regarded as novel non-invasive biomarkers that may improve BD diagnosis and prognosis.

**Table:** 

Key Points *•MALAT1/miR-155 exerts potential role in Behçet’s disease.* *•MALAT1/miR-155 are promising biomarkers for Behçet’s disease.* *•MALAT1/miR-155 targets IL-6/TNF-α/CD-106 signaling.*

Key Points

*•MALAT1/miR-155 exerts potential role in Behçet’s disease.*

*•MALAT1/miR-155 are promising biomarkers for Behçet’s disease.*

*•MALAT1/miR-155 targets IL-6/TNF-α/CD-106 signaling.*

## Introduction

Behçet’s disease (BD) is a systemic vasculitis disease that exhibits deleterious effects on the whole body organs by instigating inflammation in most blood vessels [1, 2]. Its prevalence is more elevated in the Mediterranean region, the Middle, and the Far East. BD often prevails in the third decade of life and is associated with relapsing and remitting phases. The precise etiology of BD is not well defined; nevertheless, preceding evidence proposed the interaction of genetic factors, immunological dysregulation, and environmental stimulators as crucial contributors. The hallmarks of BD involve vascular inflammation, and immune system activation, often ensuing in the characteristic features of the disease. BD is accompanied by oral and genital ulcers, skin lesions, and gastrointestinal and neurological involvement [3]. Despite both sexes being susceptible to BD, males typically experience more severe episodes of the disease. To date, clinical examinations are exploited to identify BD due to scarce data on validated laboratory diagnosis index or serum biomarkers that can assist in the diagnosis of the disease; thus, advent of new molecular tools became a requisite [4].

Long non-coding RNAs (lncRNAs) and microRNAs (miRNAs) have attracted much attention as they play a significant role in gene expression control [5]. LncRNAs and miRNAs are evolving as key players specifically in the pathogenesis of inflammatory diseases. MALAT1 (metastasis-associated lung adenocarcinoma transcript 1) is a prominent lncRNA that is known to be associated with many cancer cell lines and tumors. It was found to be upregulated with oncogenic function in most cancer types, including lung [6], colorectal [7], and thyroid [8] cancers, while in other types of cancer, such as glioma [9] and endometrial carcinoma [10], MALAT-1 was found to be a tumor suppressor. Beyond this, it was verified as a potential player in a myriad of studies related to inflammation [11–13]. MALAT1 deficiency was found to promote atherosclerosis and plaque inflammation [13]. It has also been revealed that MALAT1 modulates the inflammatory response in experimental autoimmune encephalomyelitis [14]. At the same time, the correlation of MALAT1 with both production of tumor necrosis factor-alpha (TNFα) in the septic cardiomyocytes model and inflammation in diabetic retinopathy has been scrutinized [15]. However, as of yet, no data exist on the potential impact of MALAT1 expression in BD and its respective correlation with the disease.

Among several miRNAs playing a promising role in controlling immunity and inflammation, miR-155 emerged as an important regulator of the immune response. Growing evidence shows that miR-155 can respond to many inflammatory stimuli, such as TNFα, interleukin-1b (IL-1β), and interferons, along with toll-like receptor (TLR) ligand [16, 17]. Simultaneously, miR-155 expression is elevated due to inflammation induction in cancer cells [18]. The repercussions of miR-155 in inflammation have been revealed to be adjusted by lncRNAs [19]. Although a recognized significant correlation between MALAT1 and miR-155 was previously stated in various pathological conditions such as atherosclerosis and hypoxia of cardiac stem cells [16, 17], the clinical relevance of this correlation in BD remains to be analyzed.

In conclusion, the expression of MALAT1 and miR-155 in BD in the Egyptian population has not been yet identified. Therefore, the current work aimed to evaluate the expression profiles of MALAT1 and miR-155, together with, the inflammatory markers; TNFα and IL-6, and the adhesion molecule, cluster of differentiation 106 (CD106), in BD patients. In addition, the study aimed to investigate the correlation of the previous markers with BD risk and activity in an attempt to allude for their possible beneficial role in BD and to improve BD diagnostics and prognosis assessment.

## Subjects and methods

### Subjects

**Fig. 1:**
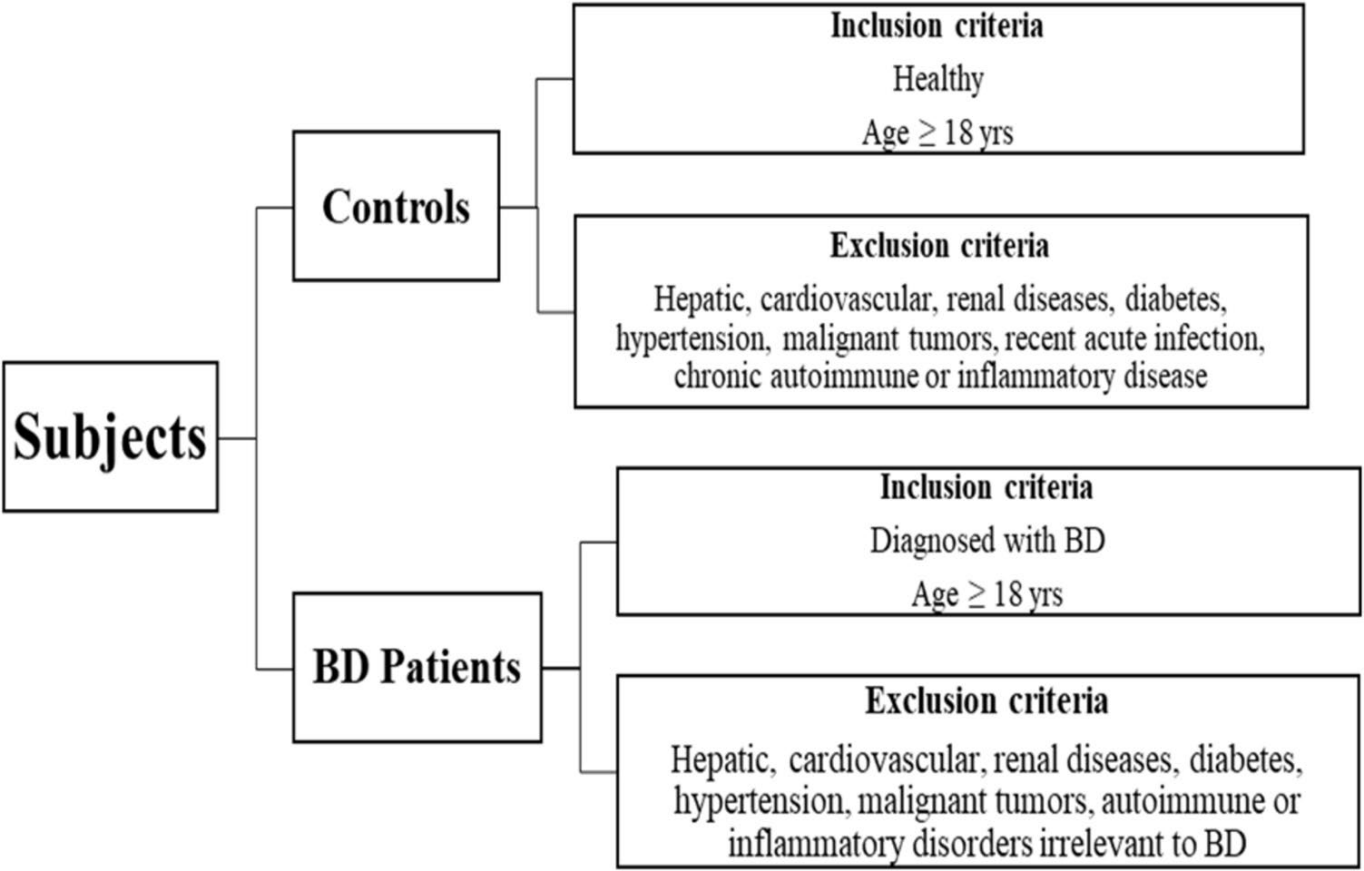
Schematic diagram for study subjects’ inclusion and exclusion criteria

The current retrospective case–control study included 74 adult Egyptian patients with BD. BD had been diagnosed according to the International Study Group for BD (ISGBD) diagnostic criteria [22]. Per this criteria, the patient must have oral (aphthous) ulcers at least three times a year along with two or more out of four symptoms: eye inflammation, genital ulcers, Pathergy reaction, and skin lesions, to be diagnosed with BD. Patients with other autoimmune or inflammatory disorders irrelevant to BD were excluded from the study. Patients with hepatic, cardiovascular, renal diseases, diabetes, hypertension, or malignant tumors were not included as well. All patients were recruited from Dermatology Outpatient Clinics and the Internal Medicine Inpatient Department of Kasr El-Aini Hospital, during a 2-year period, from May 2021 until June 2023. Fifty age- and sex-matched Egyptian healthy volunteers from analogous ethnic and socioeconomic backgrounds were enrolled in the study and served as controls. Subjects suffering hepatic, cardiovascular, renal diseases, diabetes, hypertension, malignant tumors, recent acute infection, or chronic autoimmune or inflammatory disease were not enrolled (Fig. 1).

The sample size was initially estimated before the start of the study using the G Power Software version 3.1.9.7 (http://www.gpower.hhu.de/en.html). We employed the *t* test assuming a population variance (standard deviation) of ± 0.5, a large effect size = 0.6, two independent groups, type I error *α* = 0.05, and type II error *β* = 0.2. With these assumptions, a minimum total sample size of 90 yielded a power (1 − *β*) of 80%. A total sample size of 124 yielded a power reaching 90%.

The study protocol was endorsed by the Ethical Committee of the Faculty of Pharmacy, Cairo University (reference no. BC3140). Prior to their inclusion, written consent was acquired from all subjects after a full explanation of the nature and the beneficial expectancies of the study according to the protocol approved by the local ethics committee and in accordance with the ethical standard laid down in the Helsinki declaration.

### Methods

#### Clinical assessment

Patients were put through a full medical assessment that included medical history recording and complete physical examination focusing on symptoms and signs related to BD, namely, oral, genital, skin, and joint examinations. Briefly, the BD patients who were observed to recently have at least one criterion of the ISGBD or abdominal, joint, nervous, or major blood vessel involvement in 28-day intervals were regarded to be in “the active period” of the disease, while those who had been free of any lesions for the past 4 weeks were considered as being in “the inactive phase” [23]. The patients were classified as active or inactive at the time of the study according to Behcet’s disease current activity form (BDCAF). Active BD patients received immunosuppressives (such as Azathioprine, 2–2.5 mg/kg/day; Methotrexate, 5–25 mg/week; or Cyclophosphorine A, 3–5 mg/kg/day) with corticosteroids as necessary “bridging” therapy (Prednisolone, 5–10 mg/day). While patients in the remission phase (inactive BD) did not receive any medication or were controlled on colchicine, 0.5–2 mg/day, as an anti-inflammatory agent [24].

Suspected arthritis was further confirmed by X-rays. A slit lamp examination was performed for the detection of any ocular manifestations, such as uveitis, iritis, keratitis, retinal vasculitis, or optic neuritis [25]. The Pathergy test was done by intradermal puncture under the forearm skin under sterile conditions and was considered positive if an inflammatory papule or pustule was observed within 24–48 h at the needle prick site [26]. Arterial and venous duplex scans were performed on the upper and lower extremities using B-mode and color-flow Doppler ultrasonography to detect any peripheral arterial or venous insufficiencies [27].

#### Biochemical assessment

Peripheral venous blood samples were withdrawn from all participants. The serum was separated and divided into two portions. The first one was used for lncRNA MALAT1 and miR-155 expression analysis; RNA was extracted using a miRNeasy extraction kit (Qiagen, USA). The purity and concentration of the separated RNA were assessed using a NanoDrop 2000 (Thermo Fischer Scientific, USA). RNA was reverse transcribed into cDNA using a miScript II RT Kit (Qiagen, USA). A miScript SYBR Green PCR Kit (Qiagen, USA) was used for measuring the expression of lncRNA MALAT1 and miR-155. The primer sequences utilized were as follows:

- MALAT1: forward 5′-AAAGCAAGGTCTCCCCACAAG-3′

reverse 5′-GGTCTGTGCTAGATCAAAAGGCA-3′ [28].

- miR-155: forward 5′-CTCAGACTCGGTTAATGCTAATCGTGATAGG-3′

reverse 5′-GCTGTGGCAGTGGAAGCGTGATTTATT-3′ [29].

The specificity of the used primer pairs was verified using the NCBI database. Accession numbers for MALAT1 and miR-155 are NR_144568.1 and NR_030784.1, respectively. The values of RT-PCR products were normalized by endogenous reference controls (GAPDH for MALAT1 and SNORD68 for miR-155), and the relative expression levels were calculated as 2^−∆∆Ct^.

The second serum portion was used for measurement of protein levels of TNFα, IL-6, and CD106, using ELISA kits provided by Invitrogen, Thermo Fischer Scientific, USA (Cat # BMS223HS, BMS213-2 and BMS232; respectively) according to the manufacturer’s protocols. The reactions were performed in duplicates.

### Bioinformatics analysis

RNAhybrid online tool was used to assess the complementary binding sites and the minimum free energy hybridization of MALAT1 and miR-155 https://bibiserv.cebitec.uni-bielefeld.de/rnahybrid [30]. Bioinformatics analysis was performed to investigate the gene ontology for the candidate genes. Likewise, the online database was employed to examine the potential regulations of genes of interest in a number of cellular processes and metabolic pathways http://bioinformatics.sdstate.edu/go/ [31–33].

### Statistical methods

Qualitative variables were expressed as numbers (percentage), while quantifiable data were expressed as mean ± standard deviation (SD) or median (25 − 75% percentiles) in light of data normality. Fischer exact test or chi-square test was used for the analysis of non-numeric data. Student’s *t*-test was used for analyzing parametric continuous variables. MALAT1 and miR-155 values were found to be not normally distributed according to the Kolmogorov − Smirnov normality test; thus, they were assessed using the Mann − Whitney *U* test or Kruskal − Wallis test followed by Dunn’s multiple comparisons. The diagnostic and prognostic accuracy was determined using receiver-operating-characteristic (ROC) analysis, where the area under the curve (AUC) was calculated, and optimum cutoff points were designated based on Youden’s index. Logistic regression (univariate followed by multivariate) analyses were carried out to detect predictor variables associated with BD risk and disease activity. Spearman correlation was used to measure the degree of association between different variables. The IBM SPSS Statistics for Windows, version 22 (IBM Corp., N.Y., USA) and GraphPad Prism 7.04 software (GraphPad Software Inc., USA) were used for performing the statistical analyses. Significance was declared at *P* < 0.05.

## Results

### Demographic and clinical data of BD patients

The current study included 61 male and 13 female BD patients whose ages extended from 21 to 55 years with mean age of 35.4 ± 8.3. Thirty-eight male and 12 female healthy volunteers were enrolled as controls, their ages ranged from 22 to 54 with a mean age of 34.4 ± 9.6. Patients and controls were age- and sex-matched (*P* = 0.56, *P* = 0.38; respectively). Medical history and clinical data of BD patients are presented in Table 1.

**Table 1:** Medical data of BD patients

Parameter	BD (*n* = 74)	
Duration (M)	79.1 ± 55.1	
Family history	Yes	13 (17.6%)
	No	61 (82.4%)
Disease activity	Active	52 (70.3%)
	Inactive	22 (29.7%)
Oral ulcers	Present	36 (48.6%)
	NAD	38 (51.4%)
Genital ulcers	Present	17(17.6%)
	NAD	57 (82.4%)
Ocular inflammation	Present	19 (23%)
	NAD	55 (77%)
Cutaneous lesions	Present	24 (32.4%)
	NAD	50 (67.6%)
Pathergy test	Positive	37 (50%)
	Negative	37 (50%)
Vascular insufficiency	Present	11 (14.9%)
	NAD	63 (85.1%)
Joint arthritis	Present	6 (8.1%)
	NAD	68 (91.9%)

*BD* Behçet’s disease, *M* month, *NAD* nothing abnormal detected

### Gene expression of MALAT1 and miR-155 among BD patients

**Fig. 2:**
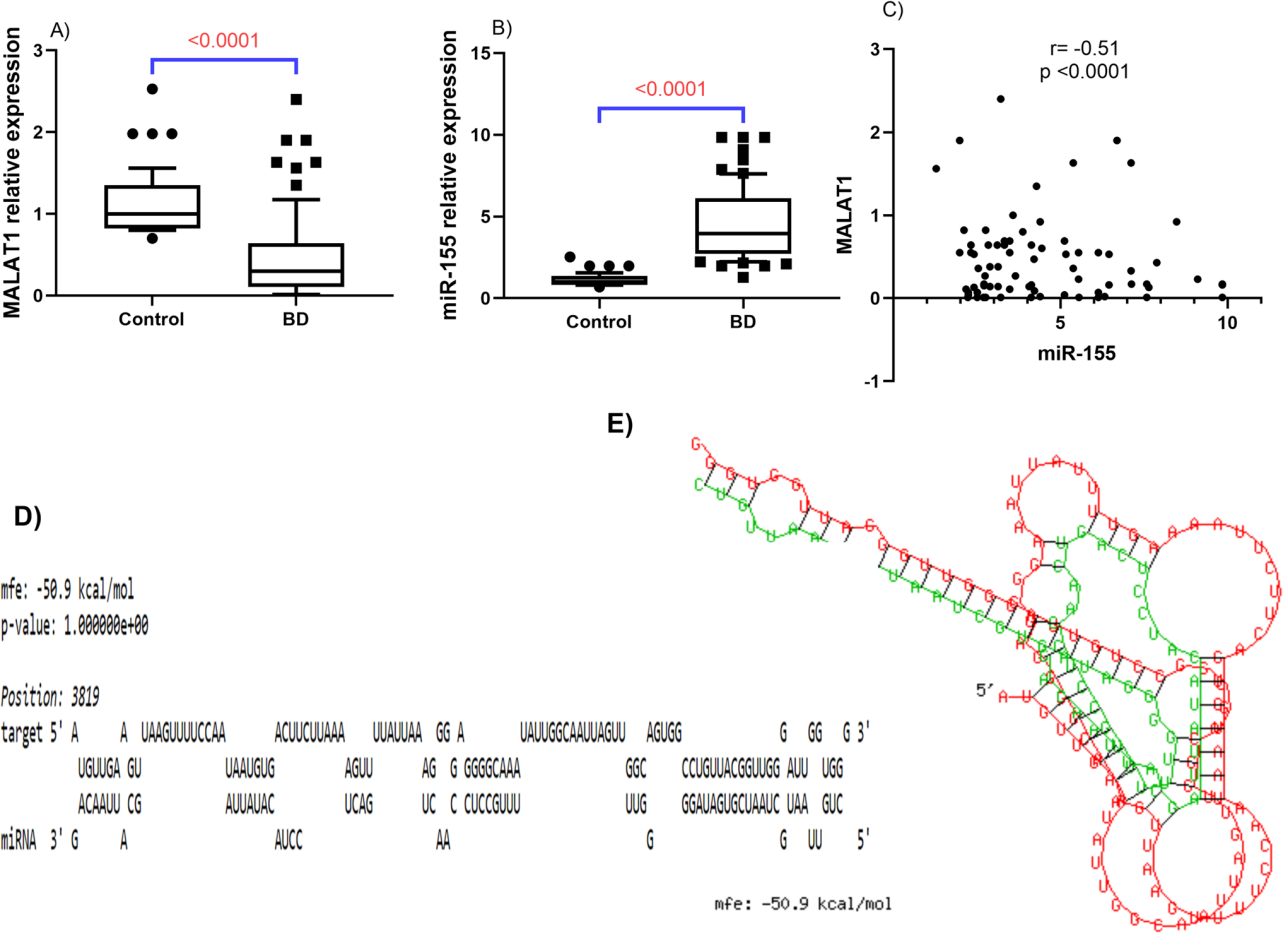
The expression levels of serum MALAT1 (**A**) and miR-155 (**B**) among BD patients relative to healthy control subjects. Data were analyzed by the Kruskal–Wallis test followed by Dunn’s multiple comparisons. Box represents the 25%–75% percentiles; the line inside the box represents the median and the upper, and lower lines represent the 10%–90% percentiles of fold change. (**C**) Spearman correlation between MALAT1 and miR-155. **D**–**E** Representation of the complementary binding sites between MALAT1 and miR-155; MALAT1 is shown in red, and miR-155 is shown in green. BD, Behçet’s disease; MALAT1, metastasis-associated lung adenocarcinoma transcript 1; mfe, minimum free energy; miR-155, micro RNA 155

As demonstrated in Fig. 2A,B, the expression of serum MALAT1 was downregulated in BD patients by 3.33-fold, relative to controls. On the flip side, miR-155 expression was upregulated by almost fourfold (*P* < 0.0001 for each). Interestingly, a strong inverse correlation was revealed between MALAT1 and miR-155 with *r* = − 0.55 and *P* < 0.0001. This association was further validated using bioinformatic analysis which showed several complementary binding sites between MALAT1 and miR-155 with mfe = − 50.9 kcal/mol as depicted in Fig. 2C–E.

### Levels of TNFα, IL-6, and CD106 among BD patients

**Fig. 3:**
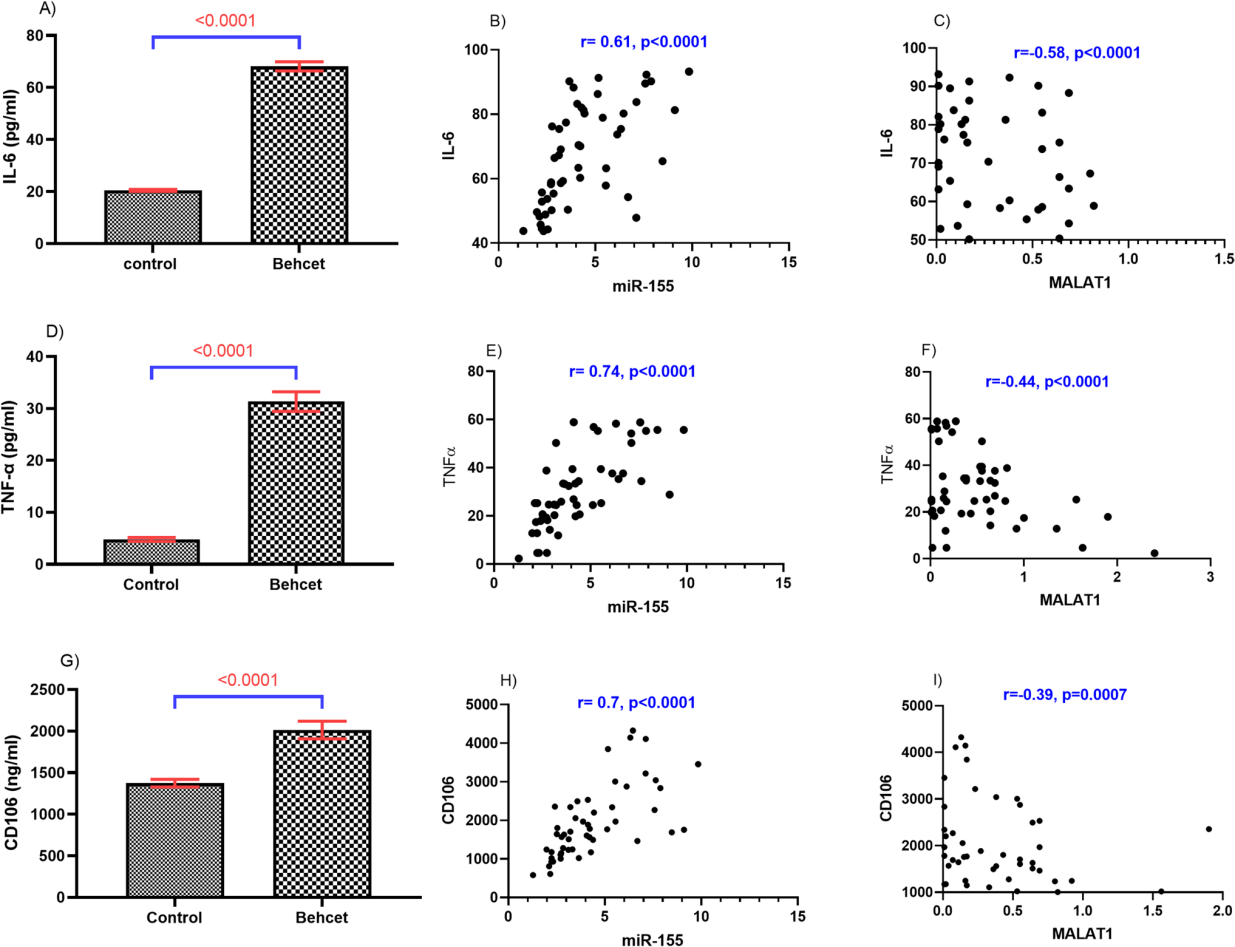
The levels of serum IL-6, TNF-α, and CD-106 among BD patients relative to healthy control subjects as well as their Spearman correlations with MALAT1 and miR-155. Data were analyzed using unpaired student t-test and represented as mean ± SE. BD, Behçet’s disease; CD106, cluster of differentiation 106; IL-6, interleukin 6; MALAT1, metastasis-associated lung adenocarcinoma transcript 1; miR-155, micro RNA 155; TNFα, tumor necrosis factor-alpha

The serum levels of the proinflammatory markers, TNFα and IL-6, were both significantly elevated in BD patients; likewise, serum CD106, a biomarker for cell adhesion, was also significantly amplified (*P* < 0.0001 for each), compared with control subjects as shown in Fig. 3. Over and beyond, a strong negative correlation was observed between MALAT1 on the one hand and miR-155, TNFα, IL-6, and CD106 on the other hand. Additionally, miR-155, TNFα, IL-6, and CD106 were all positively correlated with each other in a significant manner (Fig. 3).

### Differential expression of serum MALAT1, miR-155, and downstream targets in BD patients

**Fig. 4:**
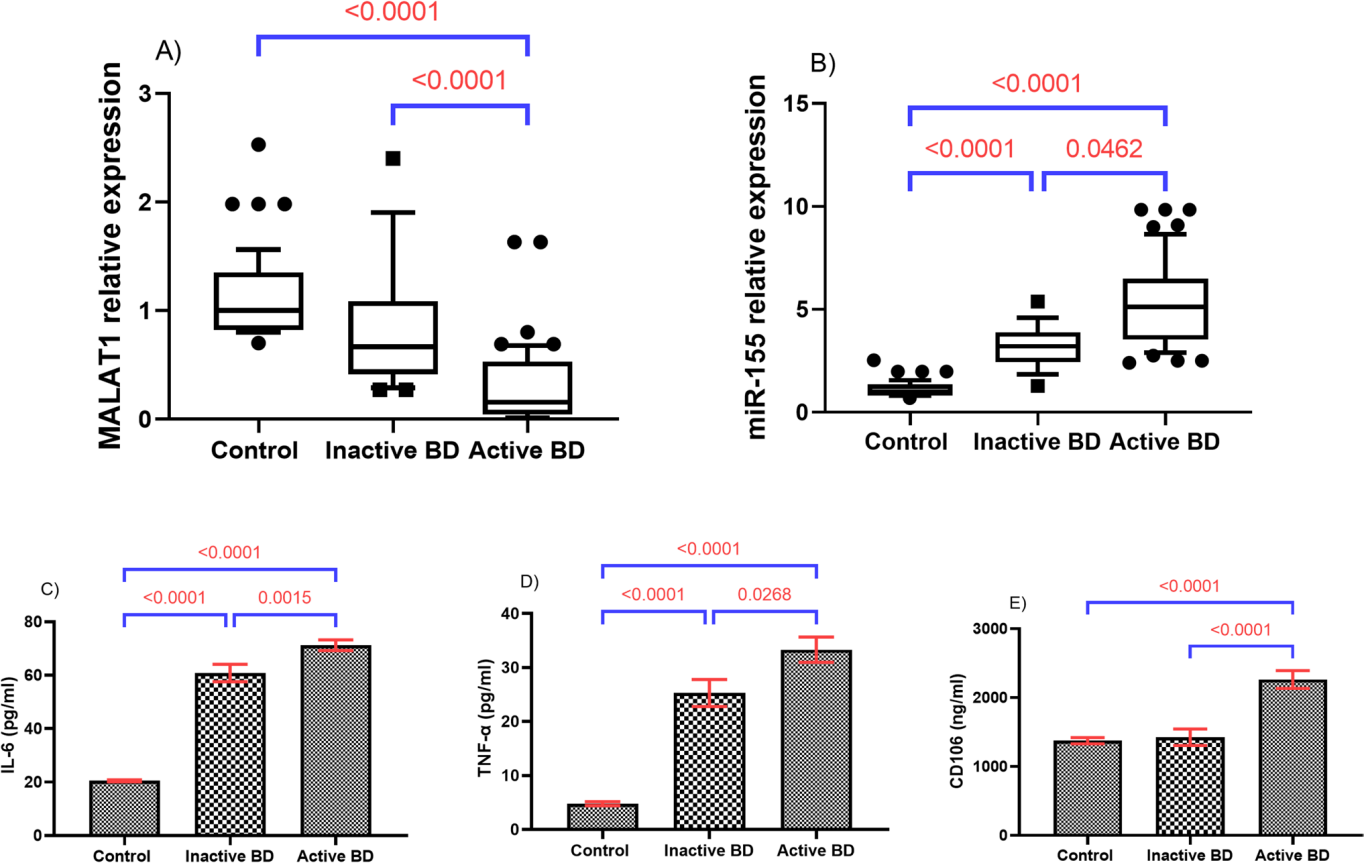
Differential expression levels of serum MALAT1 and miR-155 as well as the concentrations of IL-6, TNF-α, and CD-106 with regard to BD activity. **A**–**B** Data were analyzed by the Kruskal–Wallis test followed by Dunn’s multiple comparisons. Box represents the 25%–75% percentiles; the line inside the box represents the median and the upper, and lower lines represent the 10%–90% percentiles of fold change. Inactive BD, *n* = 22; active BD, *n* = 52. **C**–**E** Data were analyzed using one-way ANOVA followed by Tukey’s test and represented as mean ± SE. BD, Behçet’s disease; MALAT1, metastasis-associated lung adenocarcinoma transcript 1; miR-155, micro RNA 155

To investigate the differential expression of MALAT1 and miR-155 in the active and inactive states of BD, BD patients were subdivided into active and inactive groups. As illustrated in Fig. 4, results revealed a more substantial decrease in MALAT1 together with a marked elevation in miR-155 expression levels in active BD patients, relative to their inactive counterparts (*P* < 0.0001, *P* = 0.004; respectively). Besides, serum levels of TNFα, IL-6, and CD106 were markedly higher among active BD relative to inactive BD patients.

### Diagnostic and prognostic accuracy of MALAT1 and miR-155 in BD patients

**Fig. 5:**
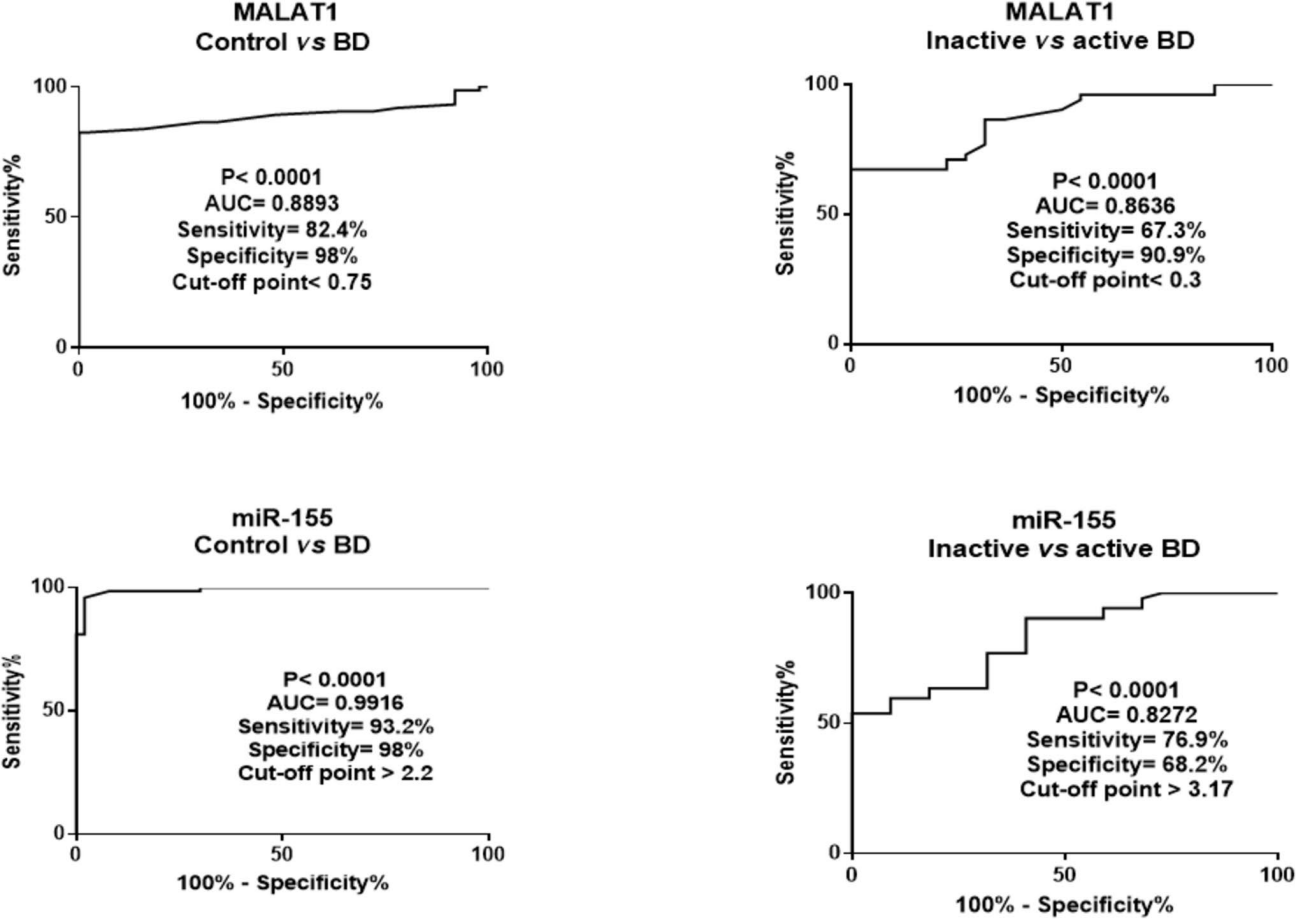
Serum MALAT1 and miR-155 as diagnostic and prognostic biomarkers for BD. Diagnostic and prognostic performances were assessed by receiver-operating-characteristic curve analysis. BD, *n* = 74; inactive BD, *n* = 22; active BD, *n* = 52. BD, Behçet’s disease; MALAT1, metastasis-associated lung adenocarcinoma transcript 1; miR-155, micro-RNA 155

ROC curve investigations (Fig. 5) revealed that serum MALAT1 could be considered a powerful marker for discriminating BD patients from healthy subjects at cutoff < 0.75 with an AUC of 0.889, optimal sensitivity and specificity of 82.4 and 98%, respectively. Furthermore, serum MALAT1 could be used as a promising indicator to distinguish BD patients in the active course of the disease from those in the inactive state at cutoff < 0.3 (AUC = 0.863, sensitivity = 67.3%, specificity = 90.9%).

As for miR-155, results indicated that it could serve as a valid biomarker for BD diagnosis at cutoff > 2.2 (AUC = 0.991, sensitivity = 93.2%, and specificity = 98%). Additionally, miR-155 could efficiently differentiate active from inactive BD patients at cutoff > 3.17 (AUC = 0.827, sensitivity = 76.9%, and specificity = 98%, 68.2%).

Intriguingly, while miR-155 was superior to MALAT1 as a diagnostic BD biomarker, MALAT1 showed more profound prognostic accuracy in discriminating patients through the active and inactive phases of BD.

### Correlation of serum MALAT1 and miR-155 levels with BD patients’ metabolic and clinicopathological data

**Fig. 6:**
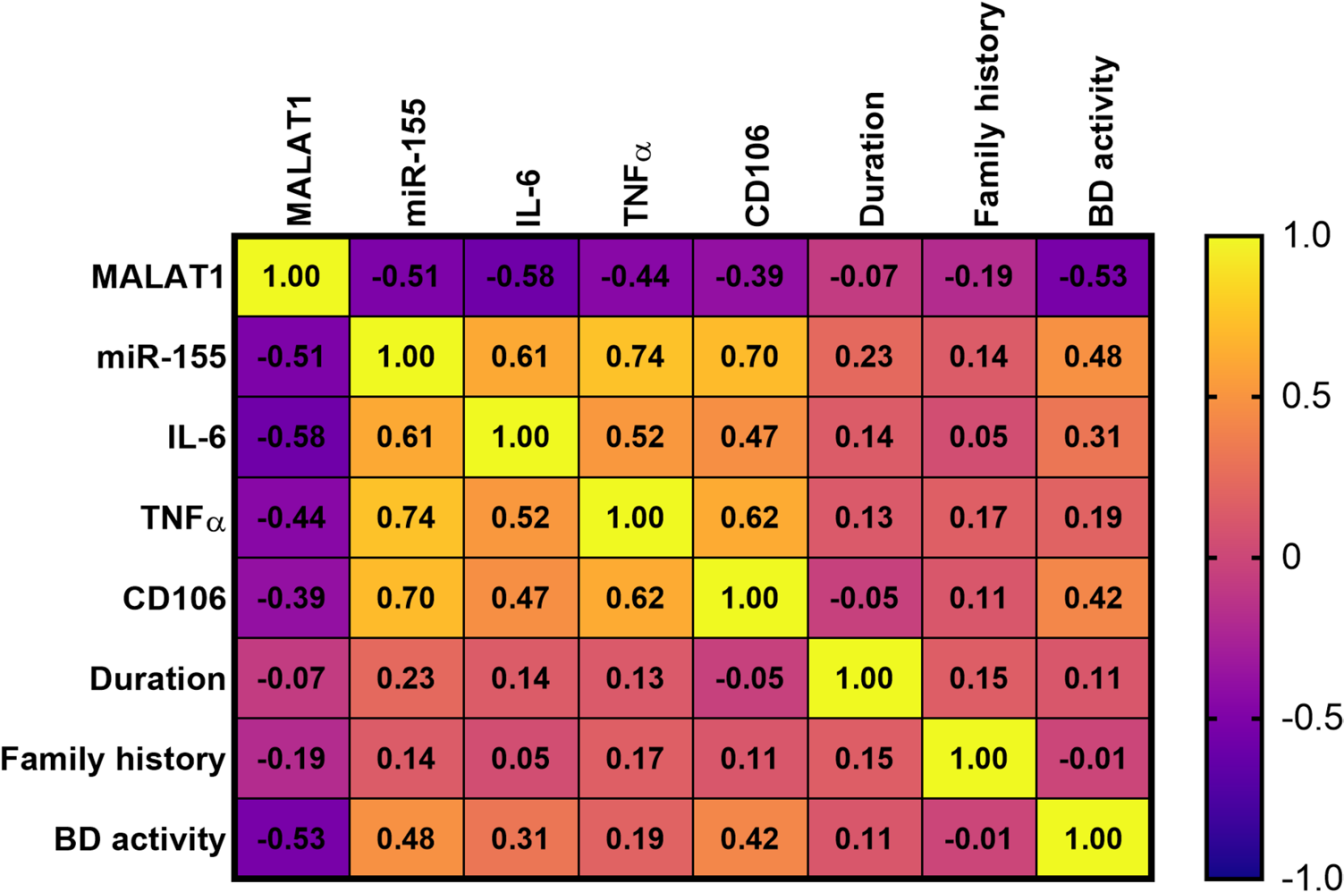
Heat map graph representing the correlations between different studied parameters among BD patients. Yellow color represents strong positive correlations (+ 1). Purple color represents strong negative correlations (− 1)

Spearman correlation analysis was conducted to unravel the extent of the connection between different biochemical and clinical data and the medical history of BD patients (Fig. 6). BD activity was remarkably correlated with all biochemical data except for TNFα. However, the family history of BD did not show any valid correlation with either biochemical parameters or clinical data. What is more, BD duration did not seem to associate with any of the studied variables.

### Logistic regression analyses to predict BD susceptibility and activity

Logistic regression analyses were performed to examine the predictive values of MALAT1, miR-155, TNFα, IL-6, and CD106 in BD risk assessment (Table 2). In the univariate analysis, serum levels of MALAT1, miR-155, TNFα, and CD106 were found to be significant predictors of BD susceptibility. However, only miR-155 was shown to be a marked independent predictor of BD in the multivariate analysis. As for BD activity prediction, serum MALAT1, miR-155, IL-6, and CD106 appeared to be remarkable predictor variables in the univariate analysis, while in the multivariate analysis, only serum MALAT1 emerged as a negative independent predictor of BD activity (Table 3).

**Table 2:** Logistic regression analysis to predict BD risk

Parameter	Coefficient	SE	*P* value	OR^a^	95% CI
Univariate analysis					
miR-155	5.2071	1.2618	** < 0.0001**	182.5687	15.5944–2165.1633
MALAT1	− 2.9513	0.5656	** < 0.0001**	0.0523	0.0173–0.1584
IL-6	2.4888	977.8690	0.9980	12.0473	0.0000
TNFα	0.4046	0.0871	** < 0.0001**	1.4987	1.2635–1.7776
CD106	0.0016	0.0004	**0.0001**	1.0016	1.0008–1.0024
Multivariate analysis					
miR-155	6.2869	3.0865	**0.0417**	537.4656	1.2679–27,832.5766
MALAT1	− 0.5950	1.0354	0.5655	0.5515	0.0725–4.1964
TNFα	1.0798	0.6153	0.0793	2.9441	0.8815–9.8333
CD106	− 0.0112	0.0069	0.1036	0.9888	0.9755–1.0023

^a^Adjusted for age and sex. *P* values in bold are statistically significant (*P* < 0.05). BD, *n* = 74; controls, *n* = 50

*BD* Behçet’s disease, *CD106* cluster of differentiation 106, *CI* confidence interval, *IL-6* interleukin 6, *MALAT1* metastasis-associated lung adenocarcinoma transcript 1, *miR-155* micro-RNA 155, *OR* odds ratio, *SE* standard error, *TNFα* tumor necrosis factor-alpha

**Table 3:** Logistic regression analysis to predict BD activity

Parameter	Coefficient	SE	*P* value	OR^a^	95% CI
Univariate analysis					
miR-155	0.9826	0.2887	**0.0007**	2.6713	1.5170–4.7038
MALAT1	−2.8664	0.8199	**0.0005**	0.0569	0.0114–0.2838
IL-6	0.0482	0.0187	**0.01**	1.0494	1.0116–1.0886
TNFα	0.0269	0.0169	0.1121	1.0273	0.9937–1.0620
CD106	0.0016	0.0005	**0.0014**	1.0016	1.0006–1.0026
Multivariate analysis					
miR-155	0.7762	0.4793	0.1053	2.1732	0.8494–5.5602
MALAT1	−2.1837	0.8828	**0.0134**	0.1126	0.0200–0.6355
IL-6	−0.0486	0.0332	0.1434	0.9525	0.8925–1.0166
CD106	0.0005	0.0008	0.4651	1.0005	0.9991–1.0020

^a^Adjusted for age and sex. *P* values in bold are statistically significant (*P* < 0.05). Active BD, *n* = 52; inactive BD, *n* = 22

*BD* Behçet’s disease, *CD106* cluster of differentiation 106, *CI* confidence interval, *IL-6* interleukin 6, *MALAT1* metastasis-associated lung adenocarcinoma transcript 1, *miR-155* micro-RNA 155, *OR* odds ratio, *SE* standard error, *TNFα* tumor necrosis factor-alpha

### Gene ontology investigations

**Fig. 7:**
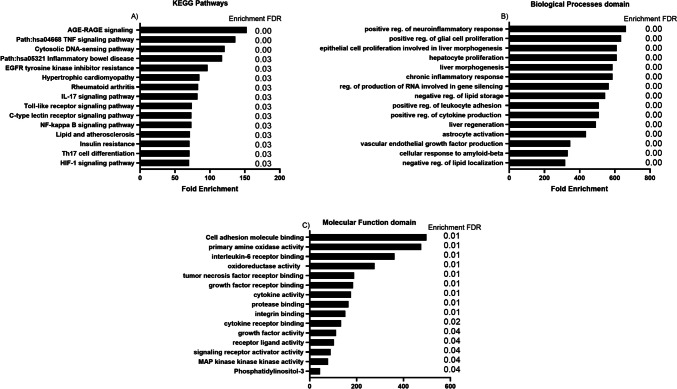
Gene ontology analysis of the studied genes. **A** Data relevant to KEGG pathways. **B** Biological processes. **C** Molecular functions. Data are sorted based on fold enrichment

Gene ontology and the Kyoto Encyclopedia of Genes and Genomes (KEGG) were used to evaluate the involvement of the studied genes in diverse signaling pathways and molecular processes (Fig. 7). The investigations showed that MALAT1 and miR-155 could participate in AGE-RAGE and TLR signaling pathways, Th17 cell differentiation, and others.

## Discussion

BD is classified as a multi-systemic vasculitis with unknown etiology. The disease is characterized by recurrent various lesions and ulcers together with broadly heterogeneous clinical features. Thus far, there is no definite laboratory diagnosis of BD, but the diagnosis relies mainly on clinical features, which makes the disease easily missed or misdiagnosed. Accordingly, pursuing novel disease markers became a demand for improving the diagnosis and prognosis of BD. Lately, more focus has been shed on the emerging role of cellular ncRNAs including specific lncRNAs and miRNAs in the regulation of autoimmune inflammatory diseases through modulating gene expression [34, 35]. MALAT1 is a lncRNA that has been reported to act as an inflammatory regulator [36]. Still, there are no reports relating MALAT1 to the incidence of BD or the activity of the disease. Regarding BD as an inflammatory disease, an altered expression of MALAT1 was expected to occur in BD patients. In the current study, a noticeable downregulation of serum MALAT1 was found in active BD patients relative to inactive patients and controls.

This finding is in agreement with previous data showing that disruption in MALAT1 expression has been identified as a culprit in the pathology of inflammatory diseases. Firstly, the contribution of MALAT1 to neuroinflammation in CNS tissues from multiple sclerosis (MS) patients was verified. MALAT1 was found to be downregulated at the peak of the disease which resulted in aggravation of neuroinflammation through disturbing macrophage and T cell differentiation [14]. Secondly, it was demonstrated that MALAT1 was significantly downregulated in human plaques versus normal arteries, a condition that augmented atherosclerotic lesion formation and elaborated the anti-inflammatory role of MALAT1 as a critical regulator of atherosclerosis via controlling different inflammatory mediators [13].

MiR-155 has received attention as a crucial participant in immune response and is nominated as a hallmark in inflammatory diseases. Herein, we observed, for the first time, a marked upregulation in miR-155 expression level in active BD patients relative to the inactive patients and control subjects. Notably, its level was also elevated in patients with inactive BD relevant to controls. This finding is consistent with previous evidence showing significantly high levels of miR-155 in peripheral blood mononuclear cells (PBMCs) of BD patients. This miR-155 elevated expression was interrelated with the downregulation of cytotoxic T lymphocyte-associated antigen-4 (CTLA-4) that possesses a suppressing role affecting the priming phase of the immune response. Remarkably, expression of miR-155 was found to be higher in BD patients with uveitis and phlebitis [3].

Counter to our findings, previous data by Ahmadi et al. (2019) showed low levels of miR-155 in Iranian BD patients relative to controls. This down expression caused a declined level of regulatory T (Treg) cells which perform crucial functions in protection against autoimmune diseases [37]. Moreover, Liang et al. (2021) disclosed that the expression of miR-155 declined in dendritic cells (DCs) from active Chinese BD patients [38]. Eventually, this controversy in results might be imputable to several factors such as; predisposing genetic variation which contributes to a discrepancy in disease development among different populations, changes in sample size, or variations in gene expression patterns in different tissue types included in the studies.

As a well-defined regulatory mechanism, lncRNAs can interfere with miRNA cascades by competing for miRNA response elements [39], and thus act as a natural miRNA sponge reducing the binding of endogenous miRNAs to target genes. Previous data showed that the binding sites of MALAT1 and miR-155 were predicted by using starBase software and it was concluded that MALAT1 could negatively modulate the expression of miR-155 by sponging it [40]. In context, the present investigation detected a marked inverse link between MALAT1 and miR-155 that was anchored by bioinformatics analysis results which revealed several complementary binding sites between MALAT1 and miR-155 [41]. In support of our findings, the potential association between MALAT1 and miR-155 concerning several pathologic conditions was previously investigated. Earlier, it has been reported that MALAT1 may have a role in the etiology of atherosclerosis by acting as an influential atherosclerosis repressor via sponging miR-155 in human coronary artery endothelial cells (HCAECs) [20]. In addition, the link between MALAT1 and miR-155 was explored in the CoCl_2_-induced cardiac stem cells (CSCs) hypoxia model, where repression of MALAT1 led to increased miR-155 expression that sequentially, hindered cell proliferation and migration in CSCs [21]. Worth noting, both serum MALAT1 and miR-155, herein, served as excellent discriminators of active BD patients from the inactive ones. Notably, miR-155 was revealed as a marked independent predictor for BD risk in multivariate logistic analysis, yet, only MALAT1 was shown to be an independent negative predictor of BD activity.

Activation of innate and adaptive immune systems is believed to trigger the development of BD [42]. TNFα and IL-6, characteristic proinflammatory cytokines, are thoroughly implicated in BD aggravation, as they are released by monocytes and T lymphocytes in patients with BD uveitis, and participate in the formation of BD lesions, such as ocular lesions [43]. The current study demonstrated markedly augmented serum levels of TNFα and IL-6, as compared to control subjects. A marked difference was noticed in IL-6 levels between active and inactive BD groups. Formerly, it was elaborated that miR-155 triggers expression of macrophages and cytokines; TNFα, and IL-6 in abdominal aortic aneurysm in mice, and there was a significant positive correlation among the three of them [44]. Simultaneously, elevated interferon-γ (IFN-γ) during Staphylococcal enterotoxin B infection was found to be mediated via miR-155, highlighting a potential connection between miR-155 expression and inflammation [45].

In addition to the previous data, TNFα also upregulates the expression of a battery of different cell adhesion molecules on the surface of endothelial cells at sites of inflammation. Among these adhesion molecules, CD106 plays an essential performance in leukocyte recruitment and leukocyte adhesion through interaction with α4β1integrin. Upon adherence of α4β1integrin expressed on leukocytes to CD106 on the surface of endothelial cells, transendothelial migration of leukocytes occurs allowing activation of various inflammatory signaling pathways [46]. The expression level of CD106, herein, was markedly elevated in BD patients than in controls, and there was a significant direct relationship between CD106 level and both miR-155 and activity of the disease. In concordance, miR-155 was found to upregulate CD106 in adipose-derived mesenchymal stem cells (AD-MSCs). To further confirm the relation between proinflammatory cytokines and adhesion molecules, the current study detected significant positive correlations between TNFα, IL-6, and CD106. Interestingly, there is emerging evidence suggesting the correlation of CD106 with immunological disorders. Wang et al. (2015) disclosed that serum CD106 levels were elevated in rheumatoid arthritis relative to controls [47]. The current study sheds light on a novel crosstalk between MALAT1 and miR-155 building up a novel nRNAs circuit and their respective targets in BD.

Finally, to pave a new path for scientific inquiry, the bioinformatics section of this work demonstrated the engagement of the genes under investigation in numerous biological and molecular functions; nonetheless, future patient trials and additional research on other autoimmune disorders are still required.

One limitation of the present work is that it is a single hospital-based study; thus, selection bias was unavoidable. The study was restricted to Egyptian patients which might limit generalizing of results; therefore, further large-scale population studies are recommended to reproduce our findings. Nevertheless, we believe that the outcome of this study can be viewed as a potential approach for advanced diagnostic and therapeutic strategies for BD through targeting ncRNAs.

## Conclusion

To the best of our knowledge, this is the first study to report the possible role played by MALAT1 and miR-155 in BD pathogenesis in Egyptian patients, with reference to the disease incidence and activity, thus, emphasizing their importance for a better understanding of the disease biology and offering promising molecular diagnostic and prognostic markers, as well as, future treatment targets. Further confirmatory studies are urgently required for the unveiling of more non-coding genes that are pertinent to BD susceptibility.

## Data Availability

All data generated or analyzed during this study are included in this published article.

## References

[1] Mar S, Vb R, Lc G (2017) Behçet’s disease: review with emphasis on dermatological aspects. An Bras Dermatol 92:452–464. 10.1590/ABD1806-4841.20177359 28954091 10.1590/abd1806-4841.20177359 PMC5595589

[2] Nair JR, Moots RJ (2017) Behcet’s disease, Clinical Medicine. J R Coll Physicians Lond 17:71–77. 10.7861/clinmedicine.17-1-71 10.7861/clinmedicine.17-1-71 PMC6297594 28148585

[3] S. Kolahi, M.J. Farajzadeh, S. Alipour, A. Abhari, J. Farhadi, N. Bahavarnia, A. Malek Mahdavi, A. Khabbazi, E. Sakhinia, Determination of mir-155 and mir-146a expression rates and its association with expression level of TNF-α and CTLA4 genes in patients with Behcet’s disease, Immunology Letters 204 (2018) 55–59. 10.1016/j.imlet.2018.10.012. 10.1016/j.imlet.2018.10.012 30366049

[4] Puccetti A, Pelosi A, Fiore PF, Patuzzo G, Lunardi C, Dolcino M (2018) MicroRNA expression profiling in Behçet’s disease, J Immun Res 10.1155/2018/2405150. 10.1155/2018/2405150 PMC5964440 29854829

[5] Ko NY, Chen LR, Chen KH (2020) The role of micro rna and long-non-coding rna in osteoporosis. Int J Mol Sci 21:1–18. 10.3390/ijms21144886 10.3390/ijms21144886 PMC7402348 32664424

[6] Ji P, Diederichs S, Wang W, Böing S, Metzger R, Schneider PM, Tidow N, Brandt B, Buerger H, Bulk E, Thomas M, Berdel WE, Serve H, Müller-Tidow C (2003) MALAT-1, a novel noncoding RNA, and thymosin beta4 predict metastasis and survival in early-stage non-small cell lung cancer. Oncogene 22:8031–8041. 10.1038/SJ.ONC.1206928 12970751 10.1038/sj.onc.1206928

[7] Wu S, Sun H, Wang Y, Yang X, Meng Q, Yang H, Zhu H, Tang W, Li X, Aschner M, Chen R (2019) MALAT1 rs664589 polymorphism inhibits binding to miR-194-5p, contributing to colorectal cancer risk, growth, and metastasis. Can Res 79:5432–5441. 10.1158/0008-5472.CAN-19-0773 10.1158/0008-5472.CAN-19-0773 31311811

[8] Huang JK, Ma L, Song WH, Lu BY, Huang YB, Dong HM, Ma XK, Zhu ZZ, Zhou R (2016) MALAT1 promotes the proliferation and invasion of thyroid cancer cells via regulating the expression of IQGAP1. Biomed Pharmacother 1(83):1–7. 10.1016/J.BIOPHA.2016.05.039 10.1016/j.biopha.2016.05.039 27470543

[9] Ma KX, Wang HJ, Li XR, Li T, Su G, Yang P, Wu JW (2015) Long noncoding RNA MALAT1 associates with the malignant status and poor prognosis in glioma, Tumour Biology: The Journal of the International Society for. Oncodev Biol Med 36:3355–3359. 10.1007/S13277-014-2969-7 10.1007/s13277-014-2969-7 25613066

[10] Li Q, Zhang C, Chen R, Xiong H, Qiu F, Liu S, Zhang M, Wang F, Wang Y, Zhou X, Xiao G, Wang X, Jiang Q (2016) Disrupting MALAT1/miR-200c sponge decreases invasion and migration in endometrioid endometrial carcinoma. Cancer Lett 383:28–40. 10.1016/J.CANLET.2016.09.019 27693631 10.1016/j.canlet.2016.09.019

[11] Puthanveetil P, Chen S, Feng B, Gautam A, Chakrabarti S (2015) Long non-coding RNA MALAT1 regulates hyperglycaemia induced inflammatory process in the endothelial cells. J Cell Mol Med 19:1418–1425. 10.1111/jcmm.12576 25787249 10.1111/jcmm.12576 PMC4459855

[12] Zhao G, Su Z, Song D, Mao Y, Mao X (2016) The long noncoding RNA MALAT1 regulates the lipopolysaccharide-induced inflammatory response through its interaction with NF-κB. FEBS Lett 590:2884–2895. 10.1002/1873-3468.12315 27434861 10.1002/1873-3468.12315

[13] Cremer S, Michalik KM, Fischer A, Pfisterer L, Jaé N, Winter C, Boon RA, Muhly-Reinholz M, John D, Uchida S, Weber C (2019) Hematopoietic deficiency of the long noncoding RNA malat1 promotes atherosclerosis and plaque inflammation. Circulation. 139(10):1320–34. 10.1161/CIRCULATIONAHA.117.029015 30586743 10.1161/CIRCULATIONAHA.117.029015

[14] Masoumi F, Ghorbani S, Talebi F, Branton WG, Rajaei S, Power C, Noorbakhsh F (2019) Malat1 long noncoding RNA regulates inflammation and leukocyte differentiation in experimental autoimmune encephalomyelitis. J Neuroimmunol 328:50–59. 10.1016/j.jneuroim.2018.11.013 30583215 10.1016/j.jneuroim.2018.11.013

[15] Radhakrishnan R, Kowluru RA (2021) Long noncoding RNA MALAT1 and regulation of the antioxidant defense system in diabetic retinopathy. Diabetes 70:227–239. 10.2337/db20-0375 33051272 10.2337/db20-0375 PMC7881848

[16] O’Connell RM, Taganov KD, Boldin MP, Cheng G, Baltimore D (2007) MicroRNA-155 is induced during the macrophage inflammatory response. Proc Natl Acad Sci USA 104:1604–1609. 10.1073/PNAS.0610731104/SUPPL_FILE/10731FIG6.PDF 17242365 10.1073/pnas.0610731104 PMC1780072

[17] Abd-Elmawla MA, Elsabagh YA, Aborehab NM (2023) Association of XIST/miRNA155/Gab2/TAK1 cascade with the pathogenesis of anti-phospholipid syndrome and its effect on cell adhesion molecules and inflammatory mediators. Scientific Reports. 13(1):18790. 10.1038/s41598-023-45214-z 37914735 10.1038/s41598-023-45214-z PMC10620142

[18] Narayan N, Bracken CP, Ekert PG (2018) MicroRNA-155 expression and function in AML: an evolving paradigm. Exp Hematol 62:1–6. 10.1016/J.EXPHEM.2018.03.007 29601851 10.1016/j.exphem.2018.03.007

[19] Mahesh G, Biswas R (2019) MicroRNA-155: a master regulator of inflammation. J Int Cytokine Res 39(6):321–30. 10.1089/JIR.2018.0155 10.1089/jir.2018.0155 PMC6591773 30998423

[20] Li S, Sun Y, Zhong L, Xiao Z, Yang M, Chen M, Wang C, Xie X, Chen X (2018) The suppression of ox-LDL-induced inflammatory cytokine release and apoptosis of HCAECs by long non-coding RNA-MALAT1 via regulating microRNA-155/SOCS1 pathway. Nutr Metab Cardiovasc Dis 28:1175–1187. 10.1016/J.NUMECD.2018.06.017 30314869 10.1016/j.numecd.2018.06.017

[21] Wang Q, Lu G, Chen Z (2018) MALAT1 promoted cell proliferation and migration via MALAT1/miR-155/MEF2A pathway in hypoxia of cardiac stem cells. J Cell Biochem 120:6384–6394. 10.1002/jcb.27925 30362213 10.1002/jcb.27925

[22] International Study Group for Behçet’s Disease, Criteria for diagnosis of Behcet’s disease, The Lancet 335 (1990) 1078–108010.1016/0140-6736(90)92643-V 1970380

[23] Bhakta BB, Brennan P, James TE, Chamberlain MA, Noble BA, Silman AJ (1999) Behcet’s disease: evaluation of a new instrument to measure clinical activity. Rheumatology 38:728–733. 10.1093/rheumatology/38.8.728 10501420 10.1093/rheumatology/38.8.728

[24] Alibaz-Oner F, Direskeneli H (2021) Advances in the treatment of Behcet’s disease. Current Rheumatol Rep 23(6):47. 10.1007/S11926-021-01011-Z 10.1007/s11926-021-01011-z PMC8136102 34014377

[25] Kump LI, Moeller KL, Reed GF, Kurup SK, Nussenblatt RB, Levy-Clarke GA (2008) Behçet’s disease: comparing 3 decades of treatment response at the National Eye Institute. Can J Ophthalmol 43:468–472. 10.3129/I08-080 18711463 10.1139/i08-080 PMC2707493

[26] Evereklioglu C (2005) Current concepts in the etiology and treatment of Behçet disease. Surv Ophthalmol 50:297–350. 10.1016/j.survophthal.2005.04.009 15967189 10.1016/j.survophthal.2005.04.009

[27] Khilnani NM (2014) Duplex ultrasound evaluation of patients with chronic venous disease of the lower extremities. Am J Roentgenol 202:633–642. 10.2214/AJR.13.11465 24555602 10.2214/AJR.13.11465

[28] Fathy N, Kortam MA, Shaker OG, Sayed NH (2021) Long noncoding RNAs MALAT1 and ANRIL gene variants and the risk of cerebral ischemic stroke: an association study. ACS Chem Neurosci 12:1351–1362. 10.1021/acschemneuro.0c00822 33818067 10.1021/acschemneuro.0c00822

[29] Xiong Y, Chen S, Liu L, Zhao Y, Lin W, Ni J (2013) Increased serum microRNA-155 level associated with nonresponsiveness to hepatitis B vaccine. Clin Vaccine Immunol 20:1089–1091. 10.1128/CVI.00044-13 23637039 10.1128/CVI.00044-13 PMC3697454

[30] Rehmsmeier M, Steffen P, Höchsmann M, Giegerich R (2004) Fast and effective prediction of microRNA/target duplexes. Rna 10(10):1507–17. 10.1261/rna.5248604.and 15383676 10.1261/rna.5248604 PMC1370637

[31] Luo W, Brouwer C (2013) Pathview: An R/Bioconductor package for pathway-based data integration and visualization. Bioinformatics 29:1830–1831. 10.1093/bioinformatics/btt285 23740750 10.1093/bioinformatics/btt285 PMC3702256

[32] Ge SX, Jung D, Jung D, Yao R (2020) ShinyGO: A graphical gene-set enrichment tool for animals and plants. Bioinformatics 36:2628–2629. 10.1093/bioinformatics/btz931 31882993 10.1093/bioinformatics/btz931 PMC7178415

[33] Kanehisa M, Furumichi M, Sato Y, Ishiguro-Watanabe M, Tanabe M (2021) KEGG: Integrating viruses and cellular organisms. Nucleic Acids Res 49:D545–D551. 10.1093/nar/gkaa970 33125081 10.1093/nar/gkaa970 PMC7779016

[34] Mehana NA, Ghaiad HR, Hassan M, Elsabagh Y, Labib S, Abd-Elmawla MA (2022) LncRNA MEG3 regulates the interplay between Th17 and Treg cells in Behçet’s disease and systemic lupus erythematosus. Life Sci 309:120965. 10.1016/j.lfs.2022.120965 36155183 10.1016/j.lfs.2022.120965

[35] Abd-Elmawla MA, Elsamanoudie NM, Ismail MF, Hammam OA, El Magdoub HM (2024) The interplay of TapSAKI and NEAT-1 as potential modulators in gentamicin-induced acute kidney injury via orchestrating miR-22-3p/TLR4/MyD88/NF-қB/IL-1 β milieu: novel therapeutic approach of Betanin. Int Immunopharmacol 143:113577. 10.1016/j.intimp.2024.113577 10.1016/j.intimp.2024.113577 39541843

[36] Gao GC, Cheng XG, Wei QQ, Chen WC, Huang WZ (2019) Long noncoding RNA MALAT-1 inhibits apoptosis and matrix metabolism disorder in interleukin-1β-induced inflammation in articular chondrocytes via the JNK signaling pathway. J Cell Biochem 120:17167–17179. 10.1002/jcb.28977 31111559 10.1002/jcb.28977

[37] Ahmadi M, Yousefi M, Abbaspour-Aghdam S, Dolati S, Aghebati-Maleki L, Eghbal-Fard S, Khabbazi A, Rostamzadeh D, Alipour S, Shabani M, Nouri M, Babaloo Z (2019) Disturbed Th17/Treg balance, cytokines, and miRNAs in peripheral blood of patients with Behcet’s disease. J Cell Physiol 234:3985–3994. 10.1002/jcp.27207 30317557 10.1002/jcp.27207

[38] Liang L, Zhou Q, Feng L (2021) Decreased microRNA-155 in Behcet’s disease leads to defective control of autophagy thereby stimulating excessive proinflammatory cytokine production. Arthritis Res Ther 23(135):1–11. 10.1186/s13075-021-02517-8 33957967 10.1186/s13075-021-02517-8 PMC8101176

[39] Salmena L, Poliseno L, Tay Y, Kats L, Pandolfi PP (2011) A ceRNA hypothesis: the rosetta stone of a hidden RNA language? Cell 146:353–358. 10.1016/j.cell.2011.07.014 21802130 10.1016/j.cell.2011.07.014 PMC3235919

[40] Liang Z, Tang F (2020) The potency of lncRNA MALAT1/miR-155/CTLA4 axis in altering Th1/Th2 balance of asthma. Biosci Rep 40:1–14. 10.1042/BSR20190397 10.1042/BSR20190397 PMC7024843 31909418

[41] Shahin H, Belcastro L, Das J, Perdiki Grigoriadi M, Saager RB, Steinvall I, Sjöberg F, Olofsson P, Elmasry M, El-Serafi AT (2024) MicroRNA-155 mediates multiple gene regulations pertinent to the role of human adipose-derived mesenchymal stem cells in skin regeneration. Frontiers Bioeng Biotechnol 18(12):1328504. 10.3389/FBIOE.2024.1328504 10.3389/fbioe.2024.1328504 PMC10982420 38562669

[42] Tong B, Liu X, Xiao J, Su G (2019) Immunopathogenesis of Behcet’s disease. Front Immunol 10:665. 10.3389/FIMMU.2019.00665 30984205 10.3389/fimmu.2019.00665 PMC6449449

[43] Sugita S, Kawazoe Y, Imai A, Yamada Y, Horie S, Mochizuki M (2012) Inhibition of Th17 differentiation by anti-TNF-alpha therapy in uveitis patients with Behcet’s disease. Arthr Res Ther 14:1–2. 10.1186/AR3824 22546542 10.1186/ar3824 PMC3446476

[44] Zhang Z, Liang K, Zou G, Chen X, Shi S, Wang G, Zhang K, Li K, Zhai S (2018) Inhibition of miR-155 attenuates abdominal aortic aneurysm in mice by regulating macrophage-mediated inflammation. Bioscience reports. 38(3):BSR20171432. 10.1042/BSR20171432 10.1042/BSR20171432 PMC5938419 29459426

[45] Rao R, Nagarkatti P, Nagarkatti M (2014) Staphylococcal enterotoxin B-induced microRNA-155 targets SOCS1 to promote acute inflammatory lung injury. Infect Immun 82:2971–2979. 10.1128/IAI.01666-14 24778118 10.1128/IAI.01666-14 PMC4097622

[46] Kong DH, Kim YK, Kim MR, Jang JH, Lee S (2018) Emerging roles of vascular cell adhesion molecule-1 (VCAM-1) in immunological disorders and cancer. Int J Molecul Sci 19(4):1057. 10.3390/IJMS19041057 10.3390/ijms19041057 PMC5979609 29614819

[47] Wang L, Ding Y, Guo X, Zhao Q (2015) Role and mechanism of vascular cell adhesion molecule-1 in the development of rheumatoid arthritis. Exp Ther Med 10:1229–1233. 10.3892/ETM.2015.2635 26622470 10.3892/etm.2015.2635 PMC4533143

